# Simultaneous selection on vegetative and reproductive phenology in a perennial herb

**DOI:** 10.1002/ece3.8610

**Published:** 2022-02-15

**Authors:** Elsa Fogelström, Giulia Zacchello, Johan Ehrlén

**Affiliations:** ^1^ 7675 Department of Ecology, Environment and Plant Science Stockholm University Stockholm Sweden; ^2^ 7675 Bolin Centre for Climate Research Stockholm University Stockholm Sweden; ^3^ Department of Ecology and Genetics, Plant Ecology and Evolution Uppsala University Uppsala Sweden

**Keywords:** correlational selection, flowering phenology, indirect selection, leaf‐out, opposing selection, phenotypic selection

## Abstract

The timing of different life‐history events is often correlated, and selection might only rarely be exerted independently on the timing of a single event. In plants, phenotypic selection has often been shown to favor earlier flowering. However, little is known about to what extent this selection acts directly versus indirectly via vegetative phenology, and if selection on the two traits is correlational. We estimated direct, indirect, and correlational phenotypic selection on vegetative and reproductive phenology over 3 years for flowering individuals of the perennial herb *Lathyrus vernus*. Direct selection favored earlier flowering and shorter timespans between leaf‐out and flowering in all years. However, early flowering was associated with early leaf‐out, and the direction of selection on leaf‐out day varied among years. As a result, selection on leaf‐out weakened selection for early flowering in one of the study years. We found no evidence of correlational selection. Our results highlight the importance of including temporally correlated traits when exploring selection on the phenology of seasonal events.

## Graphical abstract

In the perennial herb *Lathyrus vernus*, direct selection favored earlier flowering and shorter timespans between leaf‐out and flowering in all years. However, early flowering was associated with early leaf‐out, and selection on leaf‐out weakened selection for early flowering in one of the study years. Our results highlight the importance of including temporally correlated traits when exploring selection on the phenology of seasonal events.

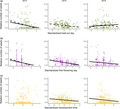

## 1 INTRODUCTION

In seasonal environments, timing of events, such as emergence, growth, reproduction, and seasonal senescence, is often temporally and developmentally correlated (Aizen, 2003; Keenan & Richardson, 2015; Kelly, 1992; O’Neil, 1997; Rathcke & Lacey, 1985; Sola & Ehrlén, 2007). Such correlations imply that selection might only rarely be exerted independently on the timing of a single event. Instead, selection on a focal phenological trait is often a combination of direct selection, and indirect selection acting via other phenological traits (Ehrlén, 2015; Galloway et al., 2018; Kelly, 1992; Rathcke & Lacey, 1985). Selection could also target the relative timing of phenological traits. Such selection has been found for development time to maturity and reproduction in both plants and animals (Kingsolver & Pfennig, 2004). Lastly, the strength or direction of selection on a focal trait might often depend on the level of other phenological traits (correlational selection; Kelly, 1992; Lande & Arnold, 1983; Phillips & Arnold, 1989). Taken together, this implies that to accurately estimate selection acting on a focal phenological trait, it is necessary to account for indirect selection via temporally correlated traits, as well as for correlational selection.

In temperate plants, the timing of reproduction is a particularly important life‐history trait, as it influences interactions with the biotic and abiotic environment and is often strongly linked to fitness (e.g., Austen et al., 2017; Ehrlén, 2015; Elzinga et al., 2007). In many environments, we expect that there is an optimal flowering time, for example, due to competition with other species for pollinators, and thus stabilizing selection on flowering phenology (Austen et al., 2017). Yet, phenotypic selection has been found to favor early flowering in many temperate plant species (Harder & Johnson, 2009; Munguía‐Rosas et al., 2011). The consistency of this pattern has raised questions as to why apparent selection for earlier flowering is so dominant, and why selection for later flowering or nonlinear, stabilizing, selection is rarely observed (Austen et al., 2017). One suggested explanation for the observed pattern is that selection on flowering time is exerted indirectly via correlated life‐history traits and that this indirect selection is not accounted for in analyses (cf. Austen et al., 2017; Rathcke & Lacey, 1985). For example, several studies have documented correlations between flowering time and vegetative phenology (e.g., Brachi et al., 2012; Diggle, 1999; Kelly, 1992; Sola & Ehrlén, 2007; Yao & Mehlenbacher, 2000). If vegetative and reproductive spring phenology are correlated, evolution of flowering time is influenced also by selection on timing of vegetative phenology, and selection will act simultaneously on both traits. In these cases, we expect direct and indirect selection on both traits, as well as on their relationship. Yet, very few studies have simultaneously examined selection on vegetative and reproductive phenology, and quantified direct, indirect, and correlational selection (but see Kelly, 1992).

In this study, we explored the relationship between vegetative spring phenology and flowering phenology in a natural population of the perennial understory herb *Lathyrus vernus* in Sweden, and used multiple regressions to estimate phenotypic selection on these two traits. Previous studies with this system have not only found that selection favors earlier flowering in most years (Ehrlén & Münzbergová, 2009; Ehrlén & Valdés, 2020) but also that flowering time is correlated with start of shoot growth and leaf development (Sola & Ehrlén, 2007). It is thus possible that some of the observed selection on flowering time is exerted indirectly, via selection on vegetative phenology. We expected that most of the among‐year differences in the distributions of phenological traits in *L*. *vernus* are driven by climatic conditions (Ehrlén & Valdés, 2020), while within‐year differences among individuals in these traits are caused by other factors, including genetic differences. To assess selection on vegetative and reproductive spring phenology, we monitored leaf‐out day and first flowering day, and recorded individual fitness in terms of seed production, for 3 years. We addressed four specific questions: (1) How closely is vegetative and reproductive phenology correlated? (2) Is there phenotypic selection acting on vegetative and reproductive phenology? (3) If so, to what extent is selection on vegetative and reproductive phenology direct versus indirect? (4) Is there selection on the relationship between vegetative and reproductive phenology, in terms of selection for time of development between leaf‐out and flowering or correlational selection?

## 2 MATERIALS AND METHODS

### 2.1 Study system

*Lathyrus vernus* (L. Bernh) is a perennial herb that is distributed over Europe and Northwest Asia (POWO, 2019). In the study area, *L*. *vernus* is mainly found in the understory of deciduous or mixed‐deciduous forests. Each plant individual produces one to several shoots that emerge early in spring and that can reach up to 40 cm in height (Ehrlén, 2002). The leaves are pinnate and consist of two to four pairs of leaflets that unfold starting with the basalmost leaf on the shoot and the pair of leaflets closest to the stem. Each flowering individual produces 1–5 racemes, each having 1–9 flowers with the basalmost flowers opening first (Ehrlén, 2002). The racemes emerge from leaf axes and start development when the neighboring leaf unfolds. Racemes and their adjacent leaf develop simultaneously, and although flowers normally open after that leaflets have unfolded, the first flower might sometimes open before the first leaflet has unfolded. A previous study with this species showed that plants with an earlier leaf development initiate flowering earlier than plants with a later leaf development (Sola & Ehrlén, 2007). In Southeast Sweden, where this study was carried out, flowering usually starts in late April to early May. Fruits contain up to 18 ovules, and the mature seeds are dispersed ballistically about 2 months after flowering (Ehrlén, 1992). *Lathyrus vernus* reproduces only sexually. Individuals of *L*. *vernus* take at least 10–15 years from germination to the first flowering event.

The reproductive performance of *L*. *vernus* is influenced by several abiotic and biotic factors, some of which have been linked to selection on flowering time. Cold April temperatures are associated with weaker selection for early flowering, possibly because frost damage reduces the benefit of flowering early (Ehrlén & Valdés, 2020). Grazing by roe deer (*Capreolus capreolus*) favors later flowering because early‐flowering individuals experience the highest levels of damage (Fogelström & Ehrlén, 2019). *Lathyrus vernus* individuals rely on pollinators (*Bombus* sp.) for their reproduction and seed production is sometimes pollen limited (Ehrlén, 1992), suggesting that there are fitness benefits of synchronizing flowering with a high availability of bumblebees. *Bruchus atomarius* (Chrysomelidae) larvae can damage a large proportion of the seeds before dispersal in the study area. *Bruchus atomarius* seed predation is sometimes linked to the timing of flowering in *L*. *vernus*, but the relationship is generally weak and its direction varies among years (Ehrlén, 1996; Ehrlén & Münzbergová, 2009).

### 2.2 Data collection

This study was conducted in a population of *L*. *vernus* in a mixed deciduous forest at Kålsö, Sweden (58°56′N, 17°39′E), in the years 2013–2015. The total number of individuals in the study population during the study years was between 800 and 900. For this study, we only included individuals that flowered and that had not been damaged (e.g., by trampling, falling tree branches or by mollusks) before the first recording. We recorded all flowering individuals in the population from shoot emergence in spring each year. Just before the first leaves in the population started to unfold, we increased the frequency of recordings to once or twice per week and continued recordings until both leaf unfolding and flowering were terminated.

We used the estimated day of year when the first leaflet unfolded as a measure of vegetative spring phenology. Leaflets open sequentially in *L*. *vernus*, and we recorded the number of unfolded leaflets at each visit. Each individual was assigned a leaf‐out day within the interval between the recording when the first leaflet was observed to be unfolded and the recording prior. We then used the proportion of open leaflets at the first recording to estimate the most probable first day with any open leaflets within that interval. We assumed that individuals with a larger proportion of open leaflets had started to leaf out earlier than individuals with a smaller proportion, and used a linear model including the total number of leaflets and its squared term to predict the number of unfolded leaflets at the first recording (Appendix S1, Table S1). Based on the deviation of the observed proportion of unfolded leaflets from this predicted proportion, we assigned individuals to a most likely leaf‐out day within each recording interval, that is, plants with larger deviations from the predicted proportion of unfolded leaflets were assigned earlier leaf‐out dates than individuals with lower values (Appendix S1, Figure S1). We assigned a leaf‐out day to all individuals that were undamaged at leaf‐out and that had been recorded on at least one occasion before leaf‐out.

We used the day of year when the first flower opened (first flowering day) as a measure of flowering phenology. We recorded the size of the largest bud for each flowering individual at each visit up to flowering. When first flowering day occurred between two recordings, we used the size of the largest bud at the first recording to assign a first flowering day value (Appendix S2). First flowering day for individuals grazed before the first flower was observed was estimated using information about the relationship between first flowering day and bud size, day of year for the bud size observation, and aboveground volume of intact individuals (Appendix S2). We used the number of days from of leaf‐out to first flowering day as a measure of relative timing. We refer to this timespan as “development time” henceforward.

The total number of shoots and the height and diameter of the focal shoot was measured in early July in each year. We then calculated the size of each individual in terms of the aboveground volume, using the formula for the volume of a cylinder, applying this to a focal shoot, and multiplying the resulting volume with the number of shoots (aboveground volume = (0.5 × shoot diameter)^2^ × shoot height × π × number of shoots). During each visit in spring, we recorded incidences of roe deer grazing. The final height of individuals grazed before the last recording in spring was estimated from the shoot height–diameter relationship of nongrazed plants (Appendix S3). For individuals that had been grazed between the last recording in spring and the final shoot recording in early July, we used the maximum recorded height in spring as an estimate of final shoot height.

We used the number of developed seeds that had not been damaged by *B*. *atomarius* larvae, as a measure of plant fitness. We examined all fruits in the population in early July each year. The number of seeds in fruits that had not yet opened was counted in the field. Each seed was examined for *B*. *atomarius* entry holes, and then dropped to the ground within the study plot. The number of seeds in fruits that had opened prior to collection in the field was estimated at the lab at Stockholm University. In these cases, we estimated the total number of seeds from clearly visible indentations made by the seeds in the pod walls. We also registered the number of *B*. *atomarius* entry holes in the pod wall, and estimated the proportion of seeds that had been preyed upon from the relationship between the proportions of seeds preyed upon and the number of *B*. *atomarius* entrance holes in the pod wall of intact fruits. We used the relationship between these two variables established in a previous study with *L*. *vernus* to estimate seed predation (proportion of seeds preyed upon = 1−e^−1.218^ *B*. *atomarius* entry holes per seed; Appendix S3 in Fogelström & Ehrlén, 2019).

### 2.3 Statistical analyses

For the statistical analyses, we only used flowering individuals for which we were able to get estimates of size, vegetative phenology, reproductive phenology, and fitness (198, 207, and 207 individuals in 2013, 2014, and 2015, respectively). All statistical analyses were carried out in R version 4.0.5 (R Core Team, 2021). Analyses were carried out separately for each year because a large proportion of individuals (46.1%) were recorded as flowering in only 1 year, and including individual as a random effect in mixed effects models could render the models unstable (Harrison et al., 2018). Summary statistics for the variables used in the analyses are presented in Appendix S4.

To estimate the strength of the correlation between leaf‐out day and first flowering day, we calculated Pearson's *r* for the untransformed variables. We estimated phenotypic selection on leaf‐out and first flowering day using linear models (Lande & Arnold, 1983). Before analysis, we standardized leaf‐out day, first flowering day, and the development time between leaf‐out and first flowering day ($(x‐\bar{x})/\sigma$) to a mean of 0 and unit standard deviation. Aboveground volume was transformed to its natural logarithm before standardization. Fitness was relativized $\left(x/\bar{x}\right)$ to a mean of 1. For soft selection, we expect the selection surface to be related to the flowering time of a focal individual relative to other individuals within a given year, rather than to day number. For hard selection, it is possible that the selective surface is similar across years, for example, in terms of the probability of cold weather conditions, but that individuals experience different parts of this surface in different years. To examine selection corresponding to these two scenarios, we ran two sets of selection models that were based on relativizing fitness and standardizing traits within and across years, respectively (De Lisle & Svensson, 2017). These two sets of models yielded similar results, and we present the results based on local within‐year relativizations and standardizations in the main text, and the results for global across years relativizations and standardizations in Appendix S5. A relatively large proportion of the flowering individuals produced no seeds (67% in 2013, 31% in 2014, and 48% in 2015), and thus the residuals from the linear models did not meet the assumption of normality. We, therefore, evaluated the significance for the estimates from all linear models by estimating 95% bias‐corrected and accelerated (BCa) bootstrap intervals using the function “Boot” from the package “car” version 3.0–10 with 10,000 replications (Fox & Weisberg, 2019) and “boot.ci” in the package “boot” version 1.3–27 (Canty & Ripley, 2021; Davison & Hinkley, 1997) in R. Estimates for which the 95% BCa interval does not overlap 0 are considered significant at *α* = .05.

To estimate total selection on leaf‐out and first flowering day, we estimated linear selection differentials from simple linear regression models for the two traits, with fitness as a response variable and the trait as the predictor variable. We estimated nonlinear selection by adding a squared term to each model. To reduce bias of the selection estimates caused by differences in condition among plants, we included plant size as a covariate in these regression models (cf. Rausher, 1992). Below, we report the selection estimates from these models including size as “total selection.” The results of corresponding models without size are presented in Appendix S6.

To investigate to what extent selection on first flowering day was direct versus indirect via leaf‐out day, we estimated direct selection in terms of linear selection gradients for leaf‐out and first flowering day from a multiple linear regression with fitness as a response variable, and aboveground volume as a covariate. We then examined whether these estimates of direct selection differed from the estimates of total selection on leaf‐out and first flowering day. We considered estimates to differ if the estimate of direct selection was outside the BCa intervals for the estimate of total selection on each trait. Differences between the estimate of direct selection and the estimate of total selection on leaf‐out day or first flowering day were interpreted as that selection was exerted indirectly, via the other phenological trait.

We investigated selection on the relationship between leaf‐out and first flowering day in two ways: First, we estimated selection on the development time from leaf‐out to first flowering day, using linear models with relative fitness as a response variable, development time as a predictor variable, and plant size as a covariate. Second, we estimated correlational selection by including the interaction leaf‐out day × first flowering day in the multiple linear regression model with relative fitness as a response variable, leaf‐out and first flowering day as predictor variables, and with plant size as a covariate (cf. Brodie, 1992). A statistically supported effect of the leaf‐out day × first flowering day interaction indicates that there is correlational selection on the two traits, that is, the strength or direction of selection on flowering time and timing of leaf‐out is dependent on the level of the other trait. Selection on development time, on the other hand, could occur regardless of whether leaf‐out and flowering time are correlated or not, and regardless of whether selection on flowering time is dependent on the timing of leaf‐out or not.

Lastly, we ran models with leaf‐out day^2^ and first flowering day^2^ added to the model estimating correlational selection, to estimate the nonlinear selection gradients. All nonlinear selection estimates were doubled to obtain more accurate measures of the magnitude of nonlinear selection (Stinchcombe et al., 2008).

Variance inflation factors were below 2 in all cases except two, and the highest value across all analyses was 3.2. This strongly suggests that multicollinearity and separating of direct and indirect selection was not a major concern in our analyses.

## 3 RESULTS

**FIGURE 1:**
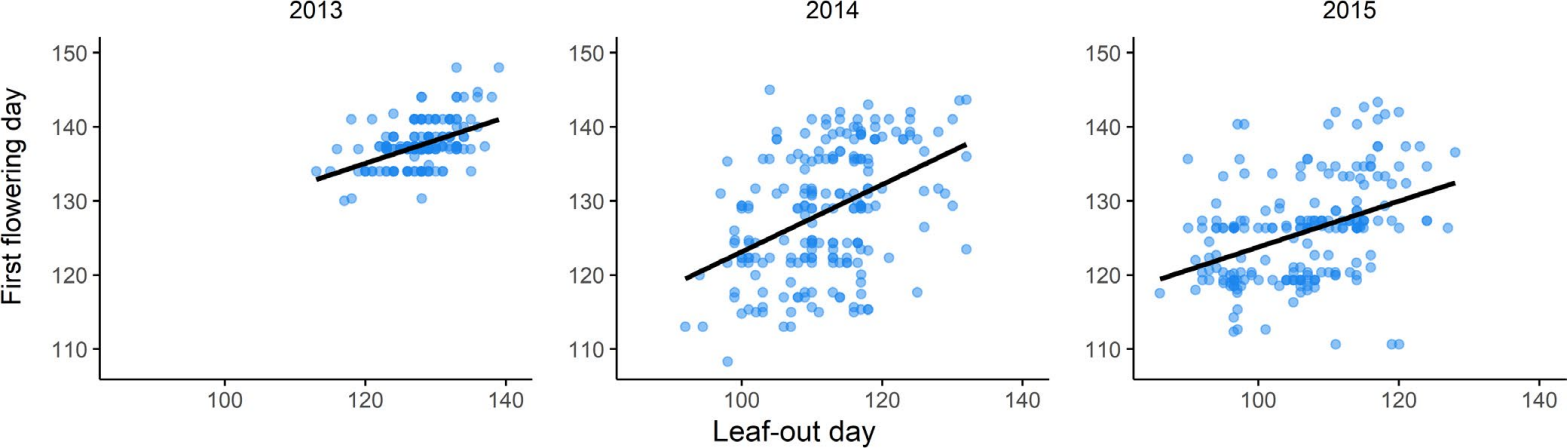
Relationship between leaf‐out day and first flowering day for *Lathyrus vernus* individuals in the years 2013, 2014, and 2015 (*n*_2013_ = 198, *n*_2014_ = 207, *n*_2015_ = 207)

During the study, individuals started to leaf‐out 23 April–20 May 2013, 2 April–14 May 2014, and 26 March–5 May 2015. Individuals initiated flowering 10–28 May in 2013, 18 April–25 May in 2014, and 20 April–23 May in 2015, respectively. The average time between leaf‐out and first flowering day was 10 days in 2013, 18 days in 2014, and 20 days in 2015. Leaf‐out and first flowering day were significantly positively correlated in all years, and correlation coefficients ranged from 0.32 to 0.46 (Pearson's correlation; 2013: *r* = .46, *t*_196_ = 7.29, *p* < .001; 2014: *r* = .41, *t*_201_ = 6.44, *p* < .001; 2015: *r* = .32, *t*_205_ = 4.77, *p* < .001; Figure 1).

**FIGURE 2:**
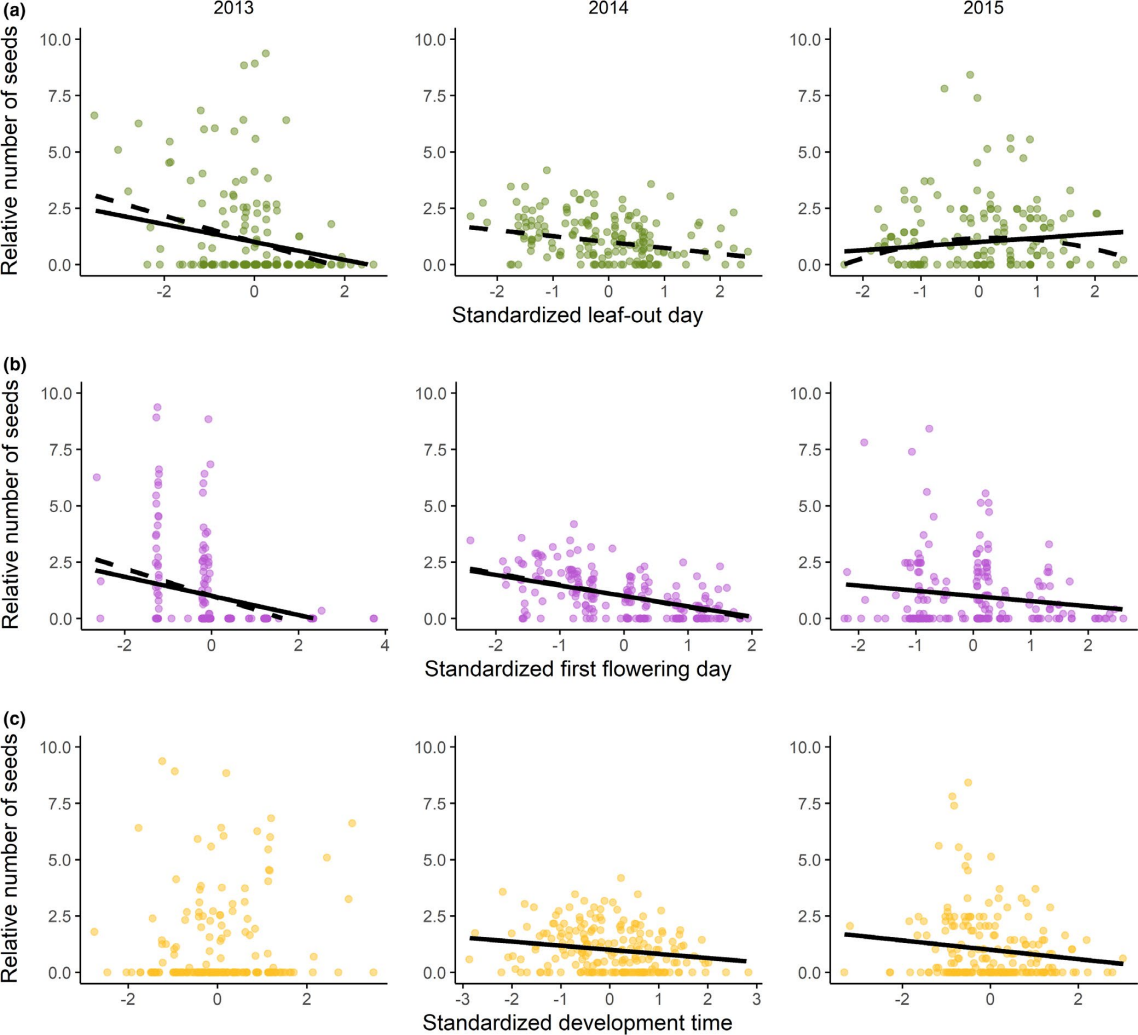
Relationships between fitness and (a) leaf‐out day, (b) first flowering day, and (c) the development time between leaf‐out and first flowering day in *Lathyrus vernus* in the years 2013 (left panels, *n* = 198), 2014 (mid‐panels, *n* = 207), and 2015 (right panels, *n* = 207). Each point represents the raw trait fitness values for one individual. Dashed lines represent total selection predicted from multiple regression models with fitness (the number of intact seeds, relativized within years) as a response variable, and (a) leaf‐out day, (b) first flowering day, or (c) the development time between leaf‐out and first flowering day as the predictor variable, and plant size (aboveground volume) included as a covariate. Solid lines represent direct selection, predicted from multiple linear regression models as above, but with both leaf‐out day and first flowering day included as predictors. All predictor variables were standardized within years to 0 mean and unit variance before analysis. Plant size was ln‐transformed before standardization

There was total selection for earlier leaf‐out in all years (Table 1a). Selection on leaf‐out day in 2015 was nonlinear, fitness being highest in individuals with an early to intermediate leaf‐out day (Table 1a; Figure 2a). There was total selection for earlier flowering in 2013 and 2014, but not in 2015 (Table 1b; Figure 2b). We found no evidence of nonlinear selection on first flowering day in any of the study years (Table 1b).

**TABLE 1:** Total selection on (a) leaf‐out day and (b) first flowering day, and direct linear, nonlinear and correlational selection for both traits (c), during three study years (*n*_2013_ = 198, *n*_2014_ = 207, *n*_2015_ = 207)

	2013			2014			2015		
	Estimate	BCa interval		Estimate	BCa interval		Estimate	BCa interval	
		Lower	Upper		Lower	Upper		Lower	Upper
(a) (Total selection: Leaf‐out day)									
Leaf‐out day	**−0.584**	**−0.816**	**−0.347**	**−0.262**	**−0.388**	**−0.135**	0.096	−0.066	0.255
Plant size	**0.767**	**0.520**	**1.111**	**0.260**	**0.134**	**0.384**	**0.278**	**0.074**	**0.554**
Leaf‐out day^2^	0.096	−0.073	0.227	0.078	−0.003	0.166	**−0.173**	**−0.347**	**−0.021**
(b) Total selection: First flowering day									
First flowering day	**−0.608**	**−0.895**	**−0.385**	**−0.502**	**−0.622**	**−0.373**	−0.155	−0.383	0.025
Plant size	**0.691**	**0.435**	**1.024**	**0.122**	**0.001**	**0.243**	0.213	−0.003	0.484
First flowering day^2^	0.109	−0.070	0.276	0.056	−0.064	0.161	−0.110	−0.242	0.157
(c) Direct selection									
Leaf‐out day	**−0.396**	**−0.659**	**−0.127**	−0.100	−0.220	0.029	**0.180**	**0.009**	**0.359**
First flowering day	**−0.425**	**−0.752**	**−0.175**	**−0.463**	**−0.589**	**−0.326**	**−0.229**	**−0.482**	**−0.046**
Plant size	**0.685**	**0.433**	**1.013**	0.112	−0.009	0.240	0.205	−0.006	0.475
Leaf‐out day^2^	0.107	−0.051	0.295	0.013	−0.072	0.110	−0.144	−0.342	0.020
First flowering day^2^	0.055	−0.159	0.308	−0.006	−0.148	0.133	−0.113	−0.256	0.160
Leaf‐out day × First flowering day	0.024	−0.322	0.320	0.129	−0.031	0.282	0.032	−0.129	0.234

Note

Model estimates with 95% bias‐corrected and accelerated (BCa) bootstrap intervals. Estimates with BCa intervals that do not overlap zero are in bold. Fitness (the response variable, the number of intact seeds) was relativized and leaf‐out day, first flowering day, and plant size (above‐ground volume) were standardized to 0 mean and unit standard deviation before analysis. Plant size was ln‐transformed before standardization. The nonlinear (quadratic) model estimates represent half of the magnitude of nonlinear selection (Stinchcombe et al., 2008).

There was direct selection for earlier leaf‐out in 2013, but in 2015 selection favored later leaf‐out instead (Table 1c; Figure 2a). There was no direct selection on leaf‐out in 2014. Direct phenotypic selection favored earlier flowering in all 3 years (Table 1c; Figure 2b). We found no support for nonlinear selection on leaf‐out or first flowering day in any of the study years.

The estimate of direct selection on leaf‐out in 2014 was just outside the BCa interval for the corresponding estimate of total selection, suggesting that selection for earlier leaf‐out 2014 acted largely via first flowering day (Table 1a,c). The estimates of total and direct selection on leaf‐out day in 2013 and 2015, and on first flowering day in all years, did not differ significantly (Table 1b; Figure 2).

Individuals with shorter development time between leaf‐out and first flowering day had higher fitness than individuals with longer development time in 2014 and 2015, but not in 2013 (Appendix S7, Table S1; Figure 2c). We found no evidence of correlational selection on leaf‐out and first flowering day in any of the three study years (Table 1c).

## 4 DISCUSSION

For understory plants in temperate regions, leafing out and flowering before canopy closure should be important for fitness (Augspurger, 2008; Ida & Kudo, 2008). Because the timing of these two events are often developmentally and temporally correlated, selection on either event cannot be accurately estimated independently (Diggle, 1999; Kelly, 1992; Lande & Arnold, 1983). Our results with the perennial herb *L*. *vernus* showed that flowering phenology was consistently correlated with vegetative phenology during spring, and that phenotypic selection was acting on both traits, as well as on development time. We also found that selection on vegetative phenology affected selection on flowering time in 1 year, but that selection favored early onset of flowering in all three study years, also when accounting for indirect selection via leaf‐out day.

Leaf‐out and first flowering day were significantly positively correlated in all 3 years, implying that flowering time is constrained by the timing of leaf‐out in *L*. *vernus*, that is, individuals must start their vegetative development early in order to flower early (cf. Diggle, 1999; Sola & Ehrlén, 2007). At the same time, the fact that correlations between leaf‐out day and first flowering day were relatively weak in all three study years suggests that there was also considerable independent variation in the two traits. Some of this variation might be attributed to differences in shoot architecture, in terms of the placement of the first inflorescence on the shoot relative to the first leaf (Diggle, 1999; Sola & Ehrlén, 2007). The moderately strong association between timing leaf‐out and flowering initiation found in this study is in accordance with the results for other herb species (e.g., Dahlgren et al., 2007; Kelly, 1992), and suggests that selection can act independently on each trait, as well as on relative timing.

We found direct phenotypic selection on leaf‐out day in two of three years. Interestingly, selection was in opposite directions in these 2 years. Such among‐year differences in the direction of selection might be related changes in trait means due to plastic responses to interannual variation in spring temperature. In our study, selection favored earlier leaf‐out in the year when development in spring was on average latest, but favored later leaf‐out in the year when average spring development was fastest. This pattern could be the result of that early leaf‐out, relative to the population mean, implying a larger risk in years when temperatures during early spring are higher and development on average starts earlier, and that the benefits of an early development are larger in years when development on average starts later. Little is known about the agents of selection on leaf‐out time in plants, but for plants where shoot development starts in early spring it is likely that weather conditions, for example, in terms of the timing of snowmelt or late frosts events, constitute important agents of selection (cf. Augspurger, 2013; Inouye, 2008). In the alpine shrub *Salix herbacea*, selection favored intermediate leaf‐out time in sites with late snowmelt, and early leaf‐out in sites with early snowmelt (Sedlacek et al., 2015), suggesting that variation in the direction of selection was mediated by the local climate. Late spring frosts likely often mediate selection for later spring development, as suggested by a study reporting that late frost events in spring primarily damaged plants in later developmental stages (Augspurger, 2013). Interestingly, and opposite to what might be expected, direct selection for earlier leaf out was strongest in 2013, the year when mean March and April temperatures were the lowest (Average mean daily temperatures during March—2013: −2.9°C, 2014: 4.2°C, 2015: 3.7°C; and April—2013: 4.2°C, 2014: 7.0°C, 2015: 8.2°C; averages of the two nearest weather stations, Oxelösund and Södertälje; www.smhi.se). Selection on leaf‐out could also be mediated by seasonal variation in light availability, and individuals that leaf‐out early in spring before canopy closure are likely to have a fitness advantage due to a longer period of resource acquisition. Leafing out before canopy closure has been shown to be important for the growth and survival of understory tree saplings (Augspurger, 2008). Early spring phenology might also infer costs in terms of increased herbivory (Roy et al., 2004; Sedlacek et al., 2015). In *L*. *vernus*, grazing is likely to be the most intense early in the season when there are fewer alternative food sources, and it is possible that also variation in grazing intensity among years contributed to the observed variation in selection on vegetative phenology in our study (7% of individuals were grazed in 2013, 26% in 2014 and 38% in 2015).

The consistent selection for early flowering found in this study is in accordance with the results of previous studies with *L*. *vernus*, as well as with several other species (Ehrlén & Valdés, 2020; Harder & Johnson, 2009; Munguía‐Rosas et al., 2011). We did not find any evidence of stabilizing selection on flowering time, in terms of significant effects of quadratic terms in models. This suggest that potential negative effects of flowering too early, for example, due to exposure to freezing temperatures, were not important during the three study years. Selection for early flowering remained also after taking variation in vegetative phenology into account, and the results were similar for analyses that relativized fitness and standardized phenology traits within versus across years (Table 1, Appendix S5). Selection for early flowering in *L*. *vernus* has been found to be mediated by warm April temperatures (Ehrlén & Valdés, 2020). In many understory species, early flowering is likely advantageous because light availability, and possibly pollinator activity, decrease rapidly as the canopy develops (Bertin & Sholes, 1993; Ida & Kudo, 2008; McKinney & Goodell, 2010). This advantage is likely to be particularly large under warm spring conditions. Also, selection on flowering time mediated by antagonists, such as predispersal seed predators, have been shown to be important in several plant species (e.g., Kolb et al., 2007). In our study, seed predation was not correlated with either leaf‐out day (2013: *r* = .10, 2014: *r* = .05, 2015: *r* = −.11, *p* > .2 in all cases) or first flowering day or (2013: *r* = −0.05, 2014: *r* = .03, 2015: *r* = −.03, *p* > .6 in all cases), and is thus unlikely to have constituted an important selective agent.

Although it has been suggested that selection on flowering time can be mediated by correlated life‐history traits, for example, via correlations between flowering initiation and flowering duration or between flowering time and emergence time (Austen et al., 2017; Rathcke & Lacey, 1985), we are not aware of any previous study simultaneously estimating indirect selection on flowering time and leaf‐out day. In our study, there was indirect selection for early leaf‐out via start of flowering in 2014, suggesting that early flowering initiation was a main benefit of early leaf‐out in that year. We found no statistical support for indirect selection on flowering time acting via leaf‐out day, and overall, our results suggest that observed consistent selection for early flowering in *L*. *vernus* is not driven by indirect selection via the timing of leaf‐out. Still, selection for later leaf‐out might have, to some extent, counteracted and weakened selection for early flowering in one of our study years; selection for early flowering in 2015 was less than half the size of selection in the two other years. In that year, selection for early flowering was not detectable without accounting for effects of leaf‐out day. This suggests that accounting for differences in vegetative phenology can affect the ability to correctly estimate selection on flowering time. However, rather than overestimating the frequency of selection for early flowering, which we might have expected given the previous literature (e.g., Austen et al., 2017; Rathcke & Lacey, 1985), neglecting variation in leaf‐out time in our analyses would have led to underestimation of the strength of selection for early flowering.

In our study, phenotypic selection favored short development times between leaf‐out and first flowering day in the two study years with the highest mean March and April temperatures, but not in the coldest year. We are unaware of any previous studies estimating selection on the time period between leaf‐out and flowering. However, it has been hypothesized that rapid development to reproduction should be favored since it decreases the likelihood of damage before reproduction (Post et al., 2008; Williams, 1966). If this is true also in our study system, then our results suggest that this advantage of a rapid development is larger in relatively warmer springs. Still, the fitness effects of a short development time between leaf‐out day and first flowering day are difficult to separate from the effects of early flowering, and a short development time might simply be favored because it allows for early reproduction. It is also possible that selection for short development time is the result of that the optimal leaf‐out time is close to the optimal timing of flowering initiation, and that the fitness benefits of a short development time reflect independent benefits of leafing out and flowering during a particular period.

While we found selection on the relative timing of leaf‐out and first flowering day in terms of development time, we found no evidence of correlational selection on the two traits. This means that effects of each trait on fitness is independent, and that particular combinations of the two traits are not associated with a higher fitness. Our results thus imply that while a shorter development time might be beneficial for several reasons, these benefits are similar for individuals differing in leaf‐out day and first flowering day. Estimates of correlational selection are overall rare (Kingsolver et al., 2001), and for plants, we are aware of only one study reporting significant correlational selection on combinations of phenological traits (flowering time and fruit maturation, Kelly, 1992). To better understand how correlations among seasonal events affect the selection on a focal phenological trait, we therefore need studies estimating selection on combinations of seasonal events, as well as correlations and indirect selection for such events.

Our analyses of phenotypic selection provide important information about how selection can act on correlated life‐history traits. However, it is important to remember that our findings were based on female reproductive success and not on lifetime fitness, and it is possible that selection acting via male fitness differs from selection via female fitness. It is also true that trade‐offs between current and future reproduction implies that some of the advantages of early flowering observed in this study might be offset by reduced fitness in subsequent years. Still, such costs of reproduction appear to be relatively modest in *L*. *vernus*, and the probability of flowering is higher in individuals that flowered in the previous year than individuals that were nonreproductive (Ehrlén & Van Groenendael, 2001). Another potential caveat, common to most studies of phenotypic selection on flowering phenology, is that individuals that were not flowering, that is, not expressing both the traits examined, were not included in the analyses. Although we do not see any obvious reason why relationships should be different in such an “invisible fraction,” we cannot fully exclude the possibility that it might be associated with some kind of bias. Lastly, we studied simultaneous selection on leaf‐out day and first flowering day in a single population. Given that several potentially important selective agents, such as exposure to late spring frosts and pollinator availability, might vary among populations, it is likely that also the strength and direction of selection vary. Still, we believe that our qualitative conclusions regarding the correlation between timing of leaf‐out and flowering time, and the relevance of indirect selection, are generally valid.

### 4.1 Concluding remarks

To obtain accurate estimates of phenotypic selection, it is essential to consider also selection via correlated traits (Lande & Arnold, 1983). Yet, selection on the timing of life‐cycle events is rarely estimated comprehensively enough to detect indirect selection via other phenological traits or correlational selection on trait combinations. Our results show that correlations between reproductive and vegetative phenology, in combination with contrasting selection on these two traits, can affect selection on flowering phenology, although the direction of selection on flowering time remained the same in our study. More generally, the results illustrate that selection on phenological traits can only be fully understood from the perspective of the seasonal development cycle. Yet, we still know little about the relationships among temporally correlated life‐history events, and the importance of these events for fitness. Insights into the extent to which selection on the timing of seasonal events is influenced by selection on temporally correlated events are also essential to understand long‐term responses to anthropogenic climate change. This is because climate is likely to influence the phenotypic expression of sequential seasonal events as well as the optimal timing of these events (Li et al., 2016; Mohan, 2019; Zohner et al., 2018).

## CONFLICT OF INTEREST

The authors declare no conflict of interests.

## AUTHOR CONTRIBUTIONS

**Elsa Fogelström:** Conceptualization (supporting); data curation (lead); formal analysis (lead); investigation (lead); methodology (equal); writing – original draft (lead); writing – review and editing (lead). **Giulia Zacchello:** Investigation (supporting). **Johan Ehrlen:** Conceptualization (lead); data curation (supporting); formal analysis (supporting); funding acquisition (lead); investigation (supporting); methodology (equal); project administration (lead); supervision (lead); writing – original draft (supporting); writing – review and editing (supporting).

## Supporting information

Supplementary Material

## Data Availability

The datasets and code supporting this article are available at https://doi.org/10.5061/dryad.zpc866t9j.
