# Supplementary material for: Simultaneous selection on vegetative and reproductive phenology in a perennial herb
Source: Ecol Evol. 2022 Feb 15;12(2):e8610. doi: 10.1002/ece3.8610 (PMC8847147; doi:10.1002/ece3.8610)
Supplement: Supplementary file 1 — Supplementary Material [file ECE3-12-e8610-s001.docx]

# Appendices

# Appendix S1. Assigning leaf-out days

We assumed that among individuals with the same recording date for first unfolded leaflets, individuals with a large proportion of unfolded leaflets had initiated leaf-out earlier than individuals with a smaller proportion of unfolded leaflets. We based this assumption on the fact that leaves and leaflets open sequentially in *L. vernus*, and therefore, the process of leafing out takes a longer time for individuals with many leaves and leaflets than for individuals with fewer leaflets. These proportions are also influenced by the individual’s total number of leaflets and individuals with larger total number of leaflets will on average have unfolded lower proportions of leaflets than plants with fewer leaflets. Therefore, the most likely leaf-out day for each individual within the interval between recordings was estimated based on the deviation of the proportion of unfolded leaflets at the first leaf recording from the predicted proportion of unfolded leaflets given the total number of leaflets produced by the individual. The expected proportion of unfolded leaflets at the first leaf recording for each individual was obtained from linear models of the proportion of unfolded leaves on the total number of leaves, and the squared term of the total number of leaves produced during spring for each plant (Table S1). The squared term was added to the model to account for that relationships between the proportion of open leaflets and total number of leaflets might be non-linear. Each plant was assigned a most likely leaf-out day so that within the interval between two recordings, plants with a larger proportion of unfolded leaflets than predicted were assigned an earlier leaf-out date than individuals with a smaller proportion of unfolded leaflets than predicted by the model. The leaf-out days were distributed so that an equal number of individuals were assigned a leaf-out day on each day of the interval between recordings (Figure S1). Individuals for which the total number of leaves was unknown (e.g. due to grazing) were assigned an intermediate leaf-out day within the interval between recordings.

### Table S1: Coefficients used to predict proportions of unfolded leaflets for *L. vernus* individuals, from linear models with the proportion of unfolded leaflets as the response variable and the total number of leaves and its squared term as predictor variables. Separate models were constructed for the years 2013 (R^2^ = 0.33), 2014 (R^2^ = 0.39), and 2015 (R^2^ = 0.32).

|  | **estimate** | **SE** | **t** | **p** |
| --- | --- | --- | --- | --- |
| 2013 |  |  |  |  |
| Intercept | 0.477 | 0.02 | 19.88 | < 0.001 |
| Total number of leaflets | -0.051 | 0.01 | -9.06 | < 0.001 |
| Total number of leaflets^2^ | 0.002 | 0.00 | 6.04 | < 0.001 |
|  |  |  |  |  |
| 2014 |  |  |  |  |
| Intercept | 0.333 | 0.01 | 30.02 | < 0.001 |
| Total number of leaflets | -0.009 | 0.00 | -13.69 | < 0.001 |
| Total number of leaflets^2^ | 0.000 | 0.00 | 8.48 | < 0.001 |
|  |  |  |  |  |
| 2015 |  |  |  |  |
| Intercept | 0.365 | 0.01 | 27.19 | < 0.001 |
| Total number of leaflets | -0.011 | 0.00 | -11.99 | < 0.001 |
| Total number of leaflets^2^ | 0.000 | 0.00 | 7.72 | < 0.001 |


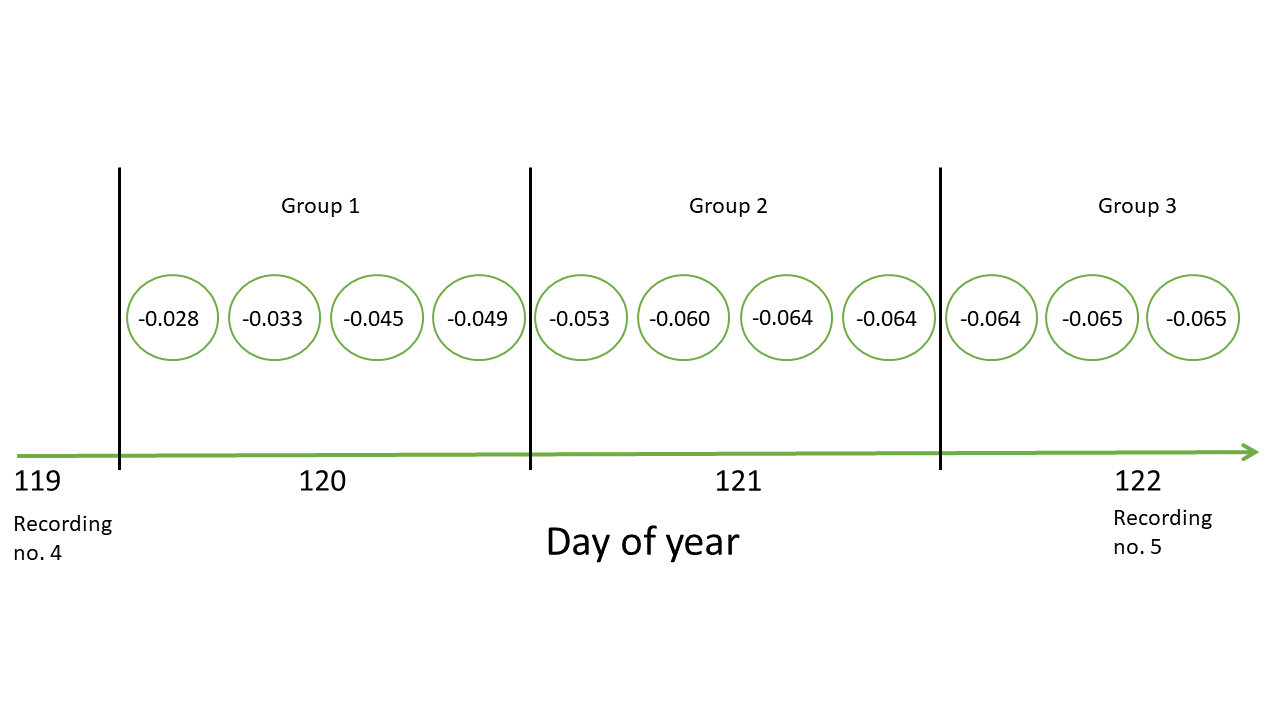
Figure S1: An example of how the method for assigning leaf-out in the *Lathyrus vernus* population, based on individuals for which the first unfolded leaflet was observed at the 5th recording (2 May) in 2013. Individuals were assigned a leaf-out day based on the deviation of their proportion of unfolded leaflets at the recording when their first unfolded leaf was observed (here recording 5) from the predicted proportion of unfolded leaflets given the total number of leaflets produced by the plant during spring. Firstly, individuals were arranged from largest to smallest deviation from the expected proportion of unfolded leaflets based on their total number of leaflets only. Each green circle in the figure represents one individual that was first observed to have unfolded leaflets at recording 5, 2013. The enclosed number is the deviation of the plant’s proportion of unfolded leaflets from their expected proportion of open leaflets. Note that in these calculations, our main aim was to estimate the order in which individuals started to leaf out. As we did not include recording date in the statistical model for 2013, all individuals that leafed out during a given recording interval could have higher or lower proportions than expected (i.e. only positive or only negative values), e.g. depending on the weather conditions during the interval. In the example all individuals had negative values, i.e. lower proportions of open leaflets than expected based on their total number of leaflets. Secondly, we separated individuals into equally sized groups as far as this was possible, with one group for each day from the previous recording up to, and including, the focal recording when the first unfolded leaflets were observed. Groups and days are delimited with vertical black lines in the figure and days are shown as day of year along the x-axis. Lastly, individuals were assigned a leaf-out day corresponding to their group’s day of year. Our focus with these calculations was to assess the order in which individuals unfolded their first leaflet during a given recording interval. We had no reason to assume that the days when the first leaflet opened were associated with our recording days, but that it was as likely to open on any day in between the recording dates as on the recording dates. The leaf-out days were therefore distributed so that an equal number of individuals were assigned a leaf-out day on each day of the interval. Differences in temperatures among days might make it more likely that the first leaflet open on some days than others. However, without actual knowledge of the specific conditions on each day, our expectation is that an equal fraction of individuals should open their first leaflet on each day during a given recording interval. Individuals for which the total number of leaves was unknown (e.g. due to grazing) were assigned an intermediate leaf-out day within the interval between two recordings.

# Appendix 2: Estimating first flowering day

Grazing occurred during different stages of floral development, resulting in four categories of plants for which first flowering day (FFD) could be estimated, either directly or from the flowering times of individuals with observed FFD, into four groups: 1) intact plants (n_2013_ = 204, n_2014_ = 186, n_2015_ = 174), 2) grazed plants for which buds had been observed prior to grazing (n_2013_ = 2, n_2014_ = 22, n_2015_ = 29), 3) plants grazed before displaying buds but after the first bud size observation in the population (n_2013_ = 3, n_2014_ = 13, n_2015_ = 25), and 4) plants grazed before displaying buds and before the first bud size observations were made in the population (n_2013_ = 2, n_2014_ = 8, n_2015_ = 15). Due to the differences between these four categories regarding the information available, different methods had to be used to estimate first flowering day for the different groups. The method used for each of the four groups is described in detail in separate sections below.

## Non-grazed plants

### Table S1. Bud size categories with description

| **Bud size, abbreviation** | **Description** |
| --- | --- |
| Small, S | Buds, but not petals, are visible on the plant |
| Medium, M | Petals are visible and have started to elongate |
| Large, L | Bud elongated, about to open |
| Very large, XL | Bud has started to open (only recorded in 2013) |

First flowering day for non-grazed plants that started flowering between recordings was estimated based on the size of the largest bud (Table S1) at the last recording before flowering. Plants with their largest bud in the last (“very large”) development stage at the recording before flowering 2013 were assigned FFD the day after the “very large” bud was observed. Plants with their largest bud in the development stage “large” were assigned FFD two thirds into the interval between the two recordings when the largest bud and the first flower were recorded. Plants with their largest bud in the development stage “medium” were assigned FFD one third into the interval between the two recordings when the largest bud and the first flower were recorded. Plants with small buds at one recording that had opened at the next recording were assigned FFD the day the first flower was observed on a plant. We will refer to the FFDs of all these plants as “observed”.

## Grazed plants with observed bud size

First flowering day of grazed plants for which bud size had been observed on at least one recording before grazing ( n_2013_ = 2, n_2014_ = 22, n_2015_ = 29) was estimated from linear models with FFD as a response variable and bud size, plant size and the day of year of the bud size observation as predictor variables for plants with observed first flowering day (i.e. the plants referred to in the previous section) using the ‘predict’ function in R. Bud size at the day of year for a given recording was the observations used for this analysis and hence multiple measurements from the same intact plant individuals were included (n_observations,2013_ = 532, n_observations,2014_ = 386, n_observations,2015_ = 411)

The correlations for observed FFD and model estimates of FFD, for plants with observed FFD in 2013, 2014, and 2015, were 0.95, 0.98, and 0.98, respectively (Pearson’s product-moment correlation, 2013: t_202_ = 41.29 p < 0.001, 2014: t_184_ = 63.09 p < 0.001, 2015: t_171_ = 63.72 p < 0.001). The observed and estimated FFDs for intact plants are presented in Figure S1. Observed FFDs for intact plants and model estimates of FFD for grazed plants are presented in Figure S2a and b, respectively.

### Table S2. Estimates from a linear model of first flowering day on bud size, plant size, and observation day of year for intact, flowering *L. vernus* individuals in the years 2013-2015 (n_observations,2013_ = 532, n_observations,2014_ = 386, n_observations,2015_ = 411) (n_2013_ = 204, n_2014_ = 186, n_2015_ = 174), (R^2^_2013_ = 0.77, R^2^_2014_ = 0.84, R^2^_2015_ = 0.81).

|  | **Estimate** | **SE** | **t-value** | **P-value** |
| --- | --- | --- | --- | --- |
| *2013* |  |  |  |  |
| Intercept | 33.245 | 2.832 | 11.739 | < 0.001 |
| Bud size (M) | 1.474 | 0.182 | 8.110 | < 0.001 |
| Bud size (L) | 3.772 | 0.186 | 20.317 | < 0.001 |
| Bud size (XL) | -1.520 | 0.177 | -8.585 | < 0.001 |
| Plant size | -0.335 | 0.076 | -4.411 | < 0.001 |
| Day of year | 0.789 | 0.020 | 39.361 | < 0.001 |
| *2014* |  |  |  |  |
| Intercept | 22.392 | 3.443 | 6.504 | < 0.001 |
| Bud size (M) | 3.227 | 0.459 | 7.024 | < 0.001 |
| Bud size (L) | 9.304 | 0.409 | 22.730 | < 0.001 |
| Plant size | -0.996 | 0.191 | -5.203 | < 0.001 |
| Day of year | 0.895 | 0.023 | 39.508 | < 0.001 |
| *2015* |  |  |  |  |
| Intercept | 22.574 | 3.344 | 6.751 | < 0.001 |
| Bud size (M) | 4.836 | 0.402 | 12.033 | < 0.001 |
| Bud size (L) | 9.893 | 0.403 | 24.565 | < 0.001 |
| Plant size | -0.412 | 0.192 | -2.140 | < 0.001 |
| Day of year | 0.859 | 0.022 | 38.882 | < 0.001 |


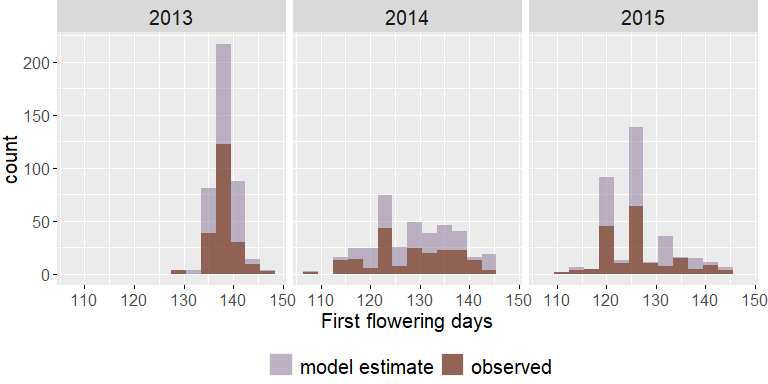


### Figure S1. Model estimates (light purple) and observed values (dark brown) of first flowering day (day of year) for *L. vernus* plants with observed first flowering day (FFD) in three years (n_2013_ = 204, n_2014_ = 186, n_2015_ = 174).

## Grazed plants without bud size

Ungulate grazers almost exclusively target *L. vernus* plants with buds or flowers [1]. Thus, plants that were grazed before buds had been recorded – but no earlier than the recording when the first buds were observed in the population – were considered to be reproductive if their estimated size was larger than the threshold size for flowering (aboveground volume of >= 230mm^3^, [2]). Our estimates of FFD for reproductive plants without bud-size observations before grazing were based on two assumptions: Firstly, we assumed that plants were in a very early stage of bud development at the recording before they were grazed, and that their largest bud would have been “small” (S) at the recording after, had they not been grazed. To describe this very early stage of bud development, we added a bud size category – “very small” (XS) – and assigned all reproductive individuals to this bud size category at the recording before the grazing event. Secondly, we assumed that the difference in average FFD between plants with XS and S buds was equal to the difference in average FFD between plants with S and “medium” (M) buds.

We let $\overline{FFD}_{bud size,rec}$ denote the average FFD of individuals with the largest bud in a given bud-size category at a given recording. Then, given our assumptions, $\overline{FFD}_{XS,rec} - \overline{FFD}_{S,rec}$ corresponds to the average of $\overline{FFD}_{M} - \overline{FFD}_{S}$, and thus, $\overline{FFD}_{XS,rec}$ can be estimated by adding the average of $\overline{FFD}_{M} - \overline{FFD}_{S}$ to $\overline{FFD}_{bud size,rec}$ of plants with the largest bud in the smallest observed bud category (i.e. $\overline{FFD}_{S,rec}$ or, if no S buds were observed during the recording; $\overline{FFD}_{M,rec}$).

To estimate FFD for plants grazed before they displayed buds, we thus first calculated $\overline{FFD}_{S}$, $\overline{FFD}_{M}$ and their difference ($\overline{FFD}_{M} - \overline{FFD}_{S}$, “Difference”, Table S3) for each recording. We then calculated the average of those differences (“Average difference”, Table S3). Lastly, we calculated $\overline{FFD}_{XS,rec}$ by adding the average difference to $\overline{FFD}_{S,rec}$ of intact plants. If no S buds were observed at the recording in question, we estimated $\overline{FFD}_{XS,rec}$ by adding the average difference to $\overline{FFD}_{M,rec}$ of intact plants. The grazed plants were then assigned an FFD equal to their estimated $\overline{FFD}_{XS,rec}$ (“Assigned FFD very small buds”, Table S3, Figure S2c).

### Table S3. Estimated (assigned) first flowering day (FFD) for reproductive *L. vernus* plants that were grazed before they displayed buds (“Assigned FFD very small buds”) in the years 2013-2015. These grazed plants were assumed to have had buds in a smallest possible bud size category (very small) at the recording before they were grazed (“Recording number”). The assigned FFD for these plants was estimated by adding the average difference (“Average difference”) in FFD to the average FFD of intact plants with “Small” buds at the same recording. If no small buds were observed, the average difference was added to the average FFD of plants with “medium” buds. Also included in the table are the date(s) for each recording, the average FFD for plants with the largest bud in the size categories “small” and “medium”, and the difference in average FFD of plants with small and medium buds at each recording, i.e. the values from which the average difference was calculated.

| **Recording number** | **Date(s)** | **Average FFD**  **Small buds**  **(**${\bar{\boldsymbol{FFD}}}_{\boldsymbol{S}}$**)** | **Average**  **FFD Medium buds**  **(**${\bar{\boldsymbol{FFD}}}_{\boldsymbol{M}}$**)** | **Difference** | **Average difference** | **Assigned FFD**  **very**  **small buds**  **(**${\bar{\boldsymbol{FFD}}}_{\boldsymbol{XS}}$**)** |
| --- | --- | --- | --- | --- | --- | --- |
| *2013* |  |  |  |  |  |  |
| 5 | 2 May | 132.0 | NA | NA | 2.8 | 134.8 |
| 6 | 6 May | 135.2 | 130.0 | 5.2 | 2.8 | 138.1 |
| 7 | 9 May | 136.8 | 134.7 | 2.1 | 2.8 | 139.6 |
| 8 | 13 May | 139.4 | 137.6 | 1.8 | 2.8 | 142.2 |
| 9 | 16 May | 141.9 | 139.7 | 2.2 | 2.8 | 144.7 |
| 10 | 20 May | NA | 144.9 | NA | 2.8 | 147.7 |
| 11 | 23 May | NA | 148.0 | NA | 2.8 | 150.8 |
| *2014* |  |  |  |  |  |  |
| 5 | 16 Apr | NA | 117.8 | NA | 5.7 | 123.4 |
| 6 | 23 Apr | 118.7 | 125.3 | 6.6 | 5.7 | 130.9 |
| 7 | 29 Apr - 30 Apr | 126.4 | 134.0 | 7.6 | 5.7 | 139.7 |
| 8 | 7 May | 132.1 | 137.9 | 5.8 | 5.7 | 143.6 |
| 9 | 13 May - 14 May | 138.7 | 141.3 | 2.6 | 5.7 | 147.0 |
| 10 | 20 May - 22 May | 145.0 | NA | NA | 5.7 | NA |
| *2015* |  |  |  |  |  |  |
| 2 | 30 Mar - 31 Mar | NA | 112.7 | NA | 5.8 | 118.5 |
| 3 | 13 Apr - 14 Apr | 112.5 | 120.5 | 8.0 | 5.8 | 126.3 |
| 4 | 20 Apr - 21 Apr | 118.2 | 123.9 | 5.7 | 5.8 | 129.7 |
| 5 | 27 Apr - 29 Apr | 125.8 | 129.8 | 4.0 | 5.8 | 135.6 |
| 6 | 4 May - 5 May | 131.2 | 137.6 | 6.4 | 5.8 | 143.4 |
| 7 | 11 May - 12 May | 138.3 | 143.3 | 5.0 | 5.8 | 149.2 |
| 8 | 18 May - 20 May | 143.3 | NA | NA | 5.8 | 149.2 |

## Plants without bud size grazed before the first bud size observations in the population

For individuals that were grazed before any flower buds were recorded in the population, the method described above could not be applied. Instead, we assumed that the development time from the XS bud category to flowering should be similar for these plants and plants with XS buds at the following recording (i.e.  that $\overline{FFD}_{XS,rec+1} - \overline{FFD}_{XS,rec}$ should correspond to $\overline{FFD}_{XS,rec+2} - \overline{FFD}_{XS,rec+1}$). Given this assumption, the $\overline{FFD}$ of plants that had been grazed before any buds had been observed in the population ($\overline{FFD}_{XS,rec}$) could be estimated by subtracting the difference in estimated $\overline{FFD}_{XS}$ for the two subsequent recordings ($\overline{FFD}_{XS,rec+2} - \overline{FFD}_{XS,rec+1}$) from $\overline{FFD}_{XS,rec+1}$. To estimate the FFD for this plant category, we thus first calculated the difference in $\overline{FFD}_{XS}$ between plants assumed to have had XS buds at the first two recordings when buds were observed in the population (i.e. $\overline{FFD}_{XS,rec+2} - \overline{FFD}_{XS,rec+1}$). We did these calculations separately for each year (diff_2013_ = 3.2, diff_2014_ = 7.5, diff_2015_ = 7.8). $\overline{FFD}_{XS}$ for plants that were first recorded as grazed when buds were visible in the population was then estimated by subtracting these differences from the estimated $\overline{FFD}_{XS}$ for plants grazed at the subsequent recording (i.e. $\overline{FFD}_{XS,rec} = \overline{FFD}_{XS,rec+1} - (\overline{FFD}_{XS,rec+2} - \overline{FFD}_{XS,rec+1}$)). The grazed plants were then assigned an FFD equal to their estimated $\overline{FFD}_{XS,rec}$ (FFD_2013_ = 131.6, FFD_2014_ = 115.9, FFD_2015_ = 110.6, Figure S2d)


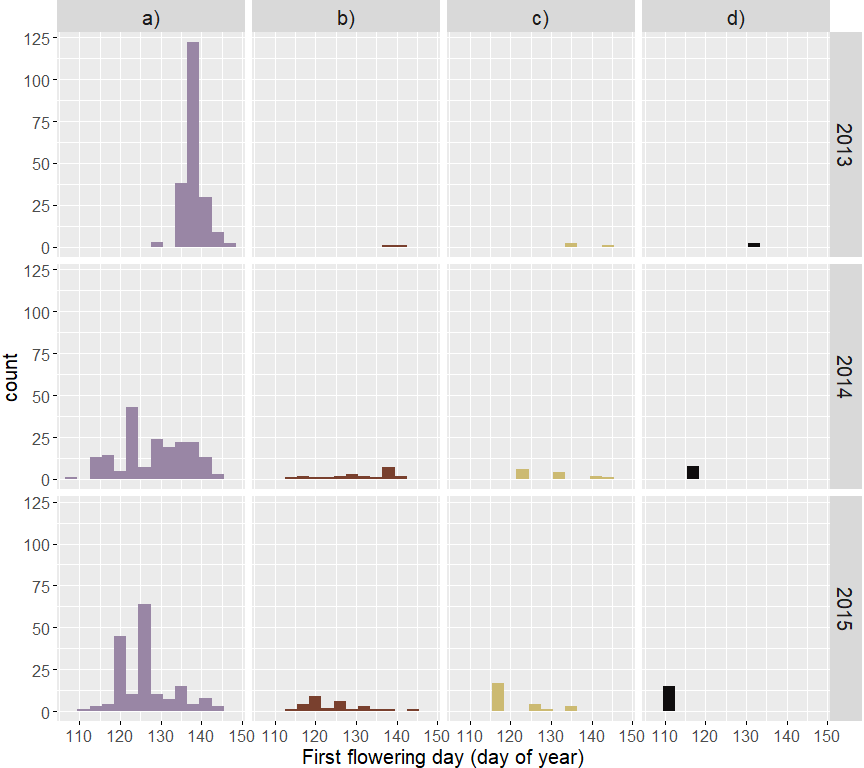


Figure S2**.** Distribution of observed (a) and estimated (b-d) first flowering days (FFD) for (a) intact plants, (b) grazed plants for which buds had been observed prior to grazing, (c) plants grazed before displaying buds but after the first buds were visible in the population, and (d) plants that had been grazed before any bud size observations had been made in the plant population, in the years 2013 (top panels) 2014 (mid-panels) and 2015 (bottom panels).

**References:**

[1] Ehrlén, J. 2002. Assessing the lifetime consequences of plant-animal interactions for the perennial herb *Lathyrus vernus* (Fabaceae). Perspectives in Plant Ecology, Evolution and Systematics 5:145–163.

[2] Ehrlén, J. 1995. Demography of the perennial herb *Lathyrus vernus*. I. Herbivory and individual performance. Journal of Ecology 83:287–295.

# Appendix S3: Estimating final shoot height of damaged plants

Individuals that had been grazed between the last recording in spring and the final recording in early July were assigned their maximum observed height from the spring recordings (n_total_ = 77, n_2013_ = 1, n_2014_ = 18, n_2015_ = 58). To estimate height for individuals that had been grazed or damaged before the final spring recording (n_total_ = 424, n_2013_ = 40, n_2014_ = 192, n_2015_ = 192), we used the measurement of shoot diameter from the last recording and estimated the coefficients from a linear model of final shoot height on shoot diameter and shoot diameter^2^ of non-grazed plants (R^2^ = 0.75, n = 1694).

Shoot height for plants without observed final shoot height was estimated by inserting the maximum observed shoot diameter of each damaged plant individual into the model formula $y=intercept+shoot diameter+shoot diameter^{2}$, where *y* is the estimated shoot height (Table S1, Figure S1). The correlation coefficient for observed and estimated measures of shoot height (mm) for intact plants was 0.87 (Pearson’s product-moment correlation, t_1688_ = 70.91, p < 0.001). The resulting shoot heights estimated from the model for grazed plants, and final heights estimated as the maximum height in spring, as well as the final height for intact plants are presented in Figure S2.

### Table S1. Coefficients from linear models of shoot height on shoot diameter of intact *Lathyrus vernus* plants (n = 1694).

|  | **Estimate** | **SE** | **t-value** | **P-value** |
| --- | --- | --- | --- | --- |
| Intercept | -2.09 | 4.65 | -0.449 | 0.654 |
| Shoot diameter | 123.76 | 5.95 | 20.790 | < 0.001 |
| Shoot diameter^2^ | -5.21 | 1.79 | -2.910 | 0.004 |


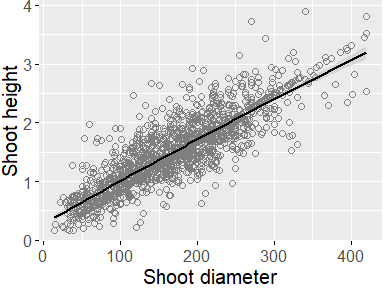


Figure S1. Height-diameter relationship from a linear model of final shoot height on shoot diameter and shoot diameter^2^ of intact *L. vernus* plants (n = 1694)


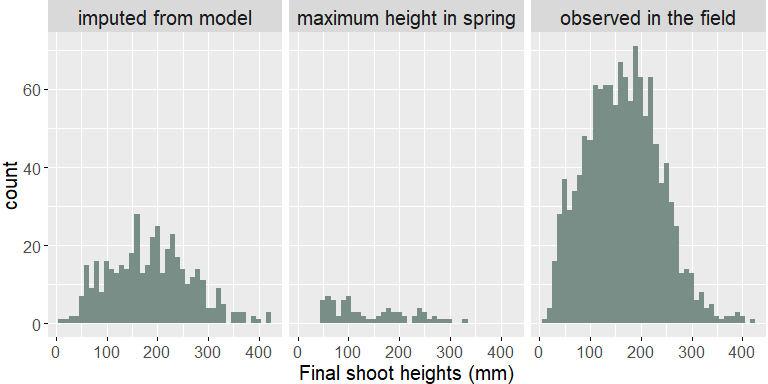


### Figure S2. Histogram of model estimates of the final height for grazed plants (left panel), final heights estimated as the maximum shoot height in spring (mid-panel) and observed values of final shoot heights (right panel) for the *L. vernus* study population (n = 1774)

# Appendix S4: Summary statistics

### Table S1. Summary statistics for the variables used for analyses of *L. vernus* plants in 2013 (n = 198), 2014 (n = 207) and 2015 (n = 207).

|  | **mean** | **sd** | **median** | **min** | **max** |
| --- | --- | --- | --- | --- | --- |
| *2013* |  |  |  |  |  |
| Final shoot height (mm) | 217.4 | 49.4 | 215.0 | 115.0 | 420.0 |
| Final shoot diameter (mm) | 1.7 | 0.4 | 1.6 | 0.6 | 2.8 |
| Plant size (aboveground volume in mm^3^) | 958.2 | 844.1 | 673.9 | 90.6 | 6334.3 |
| Leaf-out day (day of year) | 127.9 | 4.2 | 128.0 | 113.0 | 139.0 |
| First flowering day (day of year) | 137.5 | 2.8 | 137.3 | 130.0 | 148.0 |
| Development time (number of days) | 19.4 | 8.7 | 19.3 | -9.4 | 45.7 |
| Number of intact seeds | 4.9 | 7.2 | 1.0 | 0.0 | 41.0 |
| *2014* |  |  |  |  |  |
| Final shoot height (mm) | 234.9 | 58.3 | 232.0 | 92.0 | 405.0 |
| Final shoot diameter (mm) | 2.0 | 0.5 | 2.0 | 0.5 | 3.7 |
| Plant size (mm^3^) | 1590.0 | 1571.6 | 994.9 | 142.9 | 9873.2 |
| Leaf-out day (day of year) | 112.0 | 8.0 | 113.0 | 92.0 | 132.0 |
| First flowering day (day of year) | 128.6 | 8.4 | 129.0 | 108.3 | 145.0 |
| Development time (number of days) | 9.6 | 3.8 | 9.3 | -1.0 | 23.0 |
| Number of intact seeds | 1.9 | 3.7 | 0.0 | 0.0 | 18.0 |
| *2015* |  |  |  |  |  |
| Final shoot height (mm) | 219.4 | 57.6 | 220.0 | 102.0 | 417.3 |
| Final shoot diameter (mm) | 2.0 | 0.4 | 2.0 | 0.8 | 3.5 |
| Plant size (mm^3^) | 1427.5 | 1519.7 | 975.0 | 154.1 | 9873.2 |
| Leaf-out day (day of year) | 106.3 | 8.7 | 107.0 | 86.0 | 128.0 |
| First flowering day (day of year) | 125.7 | 6.8 | 126.3 | 110.6 | 143.3 |
| Development time (number of days) | 16.5 | 8.8 | 17.0 | -8.5 | 41.0 |
| Number of intact seeds | 6.9 | 6.8 | 6.0 | 0.0 | 29.0 |

#

# Appendix S5: Selection analyses using relativization of fitness and standardization of traits across all study years.

### Table S1. Selection differentials from simple linear regression models of fitness (the number of intact seeds, relativized over three years) on leaf-out day (a) or first flowering day (b), and the nonlinear estimate from that same model with the squared term added (note that the magnitude of nonlinear selection is 2 × the nonlinear model estimate, Stinchcombe et al. 2008). The model estimates are presented with 95% bias-corrected and accelerated (BCa) bootstrap intervals. Estimates with BCa intervals that do not overlap 0 are in **bold**. Fitness was relativized over the three study years and leaf-out and first flowering day were standardized over all study years to 0 mean and unit standard deviation before analysis.

|  | **2013** | | |  | **2014** | | |  | **2015** | | |
| --- | --- | --- | --- | --- | --- | --- | --- | --- | --- | --- | --- |
|  |  | **BCa interval** | |  |  | **BCa interval** | |  |  | **BCa interval** | |
|  | **Estimate** | **Lower** | **Upper** |  | **Estimate** | **Lower** | **Upper** |  | **Estimate** | **Lower** | **Upper** |
| *a) Leaf-out day* |  |  |  |  |  |  |  |  |  |  |  |
| Leaf-out day | **-0.790** | **-1.093** | **-0.505** |  | **-0.718** | **-0.986** | **-0.437** |  | 0.094 | -0.124 | 0.320 |
| Leaf-out day^2^ | 0.408 | -0.115 | 0.781 |  | 0.250 | -0.016 | 0.535 |  | **-0.308** | **-0.628** | **-0.031** |
|  |  |  |  |  |  |  |  |  |  |  |  |
| *b) First flowering day* |  |  |  |  |  |  |  |  |  |  |  |
| First flowering day | **-0.953** | **-1.324** | **-0.667** |  | **-0.806** | **-0.971** | **-0.631** |  | **-0.293** | **-0.594** | **-0.083** |
| First flowering day^2^ | 0.665 | 0.069 | 1.214 |  | 0.092 | -0.083 | 0.240 |  | -0.127 | -0.318 | 0.269 |
|  |  |  |  |  |  |  |  |  |  |  |  |

### Table S2: Total selection on (a) leaf-out day and (b) first flowering day, and direct linear, nonlinear and correlational selection for both traits (c). Model estimates with 95% bias-corrected and accelerated (BCa) bootstrap intervals. Estimates with BCa intervals that do not overlap 0 are in **bold**. Fitness (the response variable, the number of intact seeds) was relativized over all study years and leaf-out day, first flowering day, and plant size (aboveground volume) were standardized over all study years to 0 mean and unit standard deviation before analysis. Plant size was ln-transformed before standardization. The nonlinear (quadratic) model estimates represent ½ of the magnitude of nonlinear selection (Stinchcombe et al. 2008).

|  | **2013** | | |  | **2014** | | |  | **2015** | | |
| --- | --- | --- | --- | --- | --- | --- | --- | --- | --- | --- | --- |
|  |  | **BCa interval** | |  |  | **BCa interval** | |  |  | **BCa interval** | |
|  | **Estimate** | **Lower** | **Upper** |  | **Estimate** | **Lower** | **Upper** |  | **Estimate** | **Lower** | **Upper** |
| *a) Total selection: Leaf-out day* |  |  |  |  |  |  |  |  |  |  |  |
| Leaf-out day | **-0.672** | **-0.944** | **-0.415** |  | **-0.572** | **-0.845** | **-0.294** |  | 0.135 | -0.090 | 0.360 |
| Plant size | **0.349** | **0.234** | **0.502** |  | **0.376** | **0.189** | **0.556** |  | **0.300** | **0.078** | **0.600** |
| Leaf-out day^2^ | 0.306 | -0.223 | 0.714 |  | 0.249 | -0.013 | 0.523 |  | **-0.323** | **-0.649** | **-0.039** |
|  |  |  |  |  |  |  |  |  |  |  |  |
| *b) Total selection: First flowering day* |  |  |  |  |  |  |  |  |  |  |  |
| First flowering day | **-0.740** | **-1.096** | **-0.468** |  | **-0.733** | **-0.910** | **-0.546** |  | -0.197 | -0.485 | 0.027 |
| Plant size | **0.314** | **0.195** | **0.462** |  | 0.177 | -0.005 | 0.349 |  | **0.230** | **0.002** | **0.525** |
| First flowering day^2^ | 0.388 | -0.252 | 0.987 |  | 0.080 | -0.093 | 0.229 |  | -0.169 | -0.374 | 0.231 |
|  |  |  |  |  |  |  |  |  |  |  |  |
| *b) Direct selection* |  |  |  |  |  |  |  |  |  |  |  |
| Leaf-out day | **-0.455** | **-0.766** | **-0.131** |  | -0.218 | -0.494 | 0.060 |  | **0.253** | **0.022** | **0.513** |
| First flowering day | **-0.516** | **-0.940** | **-0.227** |  | **-0.676** | **-0.866** | **-0.481** |  | **-0.291** | **-0.603** | **-0.046** |
| Plant size | **0.312** | **0.197** | **0.459** |  | 0.163 | -0.016 | 0.342 |  | 0.222 | -0.008 | 0.525 |
| Leaf-out day^2^ | 0.340 | -0.169 | 0.914 |  | 0.040 | -0.231 | 0.335 |  | -0.269 | -0.622 | 0.037 |
| First flowering day^2^ | 0.194 | -0.565 | 1.134 |  | -0.009 | -0.209 | 0.189 |  | -0.174 | -0.388 | 0.232 |
| Leaf-out day × First flowering day | 0.079 | -1.101 | 1.061 |  | 0.274 | -0.061 | 0.618 |  | 0.054 | -0.229 | 0.399 |

### Table S3. Estimates of a) total phenotypic selection in terms of linear and nonlinear selection differentials and b) direct linear and nonlinear phenotypic selection in terms of gradients for the development time between leaf-out and flowering initiation in *L. vernus* with 95% bias-corrected and accelerated (BCa) bootstrap intervals. Note that the magnitude of nonlinear selection is 2 × the nonlinear model estimate (Stinchcombe et al. 2008). Development time and plant size (aboveground volume) were standardized to 0 mean and unit standard deviation, and fitness (the number of intact seeds) was relativized, over all three study years before analysis. Plant size was ln-transformed before standardization.

|  | **2013** | | |  | **2014** | | |  | **2015** | | |
| --- | --- | --- | --- | --- | --- | --- | --- | --- | --- | --- | --- |
|  |  | **BCa interval** | |  |  | **BCa interval** | |  |  | **BCa interval** | |
|  | **Estimate** | **Lower** | **Upper** |  | **Estimate** | **Lower** | **Upper** |  | **Estimate** | **Lower** | **Upper** |
| *a) Selection differentials* |  |  |  |  |  |  |  |  |  |  |  |
| Development time | 0.170 | -0.113 | 0.464 |  | **-0.336** | **-0.515** | **-0.159** |  | **-0.257** | **-0.454** | **-0.094** |
| Development time^2^ | 0.183 | -0.172 | 0.683 |  | -0.089 | -0.202 | 0.050 |  | -0.061 | -0.162 | 0.030 |
|  |  |  |  |  |  |  |  |  |  |  |  |
| *b) Selection gradients* |  |  |  |  |  |  |  |  |  |  |  |
| Development time | 0.201 | -0.055 | 0.465 |  | **-0.267** | **-0.442** | **-0.085** |  | **-0.216** | **-0.402** | **-0.052** |
| Plant size | **0.388** | **0.266** | **0.544** |  | **0.432** | **0.250** | **0.613** |  | **0.251** | **0.042** | **0.542** |
| Development time^2^ | 0.241 | -0.080 | 0.654 |  | -0.037 | -0.156 | 0.100 |  | -0.079 | -0.202 | 0.027 |

**References:** Stinchcombe, J. R., A. F. Agrawal, P. A. Hohenlohe, S. J. Arnold, and M. W. Blows. 2008. Estimating nonlinear selection gradients using quadratic regression coefficients: double or nothing? Evolution 62:2435–2440.

# Appendix S6

### Table S1. Selection differentials from simple linear regression models of fitness (the number of intact seeds, relativized) on leaf-out day (a) or first flowering day (b), and the nonlinear estimate from that same model with the squared term added (note that the magnitude of nonlinear selection is 2 × the nonlinear model estimate, Stinchcombe et al. 2008). The model estimates are presented with 95% bias-corrected and accelerated (BCa) bootstrap intervals. Estimates with BCa intervals that do not overlap 0 are in **bold**. Leaf-out and first flowering day were standardized within years before analysis.

|  | **2013** | | |  | **2014** | | |  | **2015** | | |
| --- | --- | --- | --- | --- | --- | --- | --- | --- | --- | --- | --- |
|  |  | **BCa interval** | |  |  | **BCa interval** | |  |  | **BCa interval** | |
|  | **Estimate** | **Lower** | **Upper** |  | **Estimate** | **Lower** | **Upper** |  | **Estimate** | **Lower** | **Upper** |
| *a) Leaf-out day* |  |  |  |  |  |  |  |  |  |  |  |
| Leaf-out day | **-0.686** | **-0.943** | **-0.435** |  | **-0.329** | **-0.453** | **-0.203** |  | 0.067 | -0.092 | 0.222 |
| Leaf-out day^2^ | 0.128 | -0.036 | 0.248 |  | 0.079 | -0.006 | 0.167 |  | **-0.165** | **-0.332** | **-0.013** |
|  |  |  |  |  |  |  |  |  |  |  |  |
| *b) First flowering day* |  |  |  |  |  |  |  |  |  |  |  |
| First flowering day | **-0.784** | **-1.084** | **-0.538** |  | **-0.552** | **-0.663** | **-0.435** |  | **-0.230** | **-0.471** | **-0.063** |
| First flowering day^2^ | **0.187** | **0.018** | **0.346** |  | 0.065 | -0.053 | 0.173 |  | -0.083 | -0.207 | 0.157 |
|  |  |  |  |  |  |  |  |  |  |  |  |

**References:** Stinchcombe, J. R., A. F. Agrawal, P. A. Hohenlohe, S. J. Arnold, and M. W. Blows. 2008. Estimating nonlinear selection gradients using quadratic regression coefficients: double or nothing? Evolution 62:2435–2440.

# Appendix S7: Phenotypic selection on development time

### Table S1. Estimates of a) total phenotypic selection in terms of linear and nonlinear selection differentials and b) direct linear and nonlinear phenotypic selection in terms of gradients for the development time between leaf-out and flowering initiation in *L. vernus* with 95% bias-corrected and accelerated (BCa) bootstrap intervals. Note that the magnitude of nonlinear selection is 2 × the nonlinear model estimate (Stinchcombe et al. 2008). Development time and plant size (aboveground volume) were standardized before analysis. Plant size was ln-transformed before standardization.

|  | **2013** | | |  | **2014** | | |  | **2015** | | |
| --- | --- | --- | --- | --- | --- | --- | --- | --- | --- | --- | --- |
|  |  | **BCa interval** | |  |  | **BCa interval** | |  |  | **BCa interval** | |
|  | **Estimate** | **Lower** | **Upper** |  | **Estimate** | **Lower** | **Upper** |  | **Estimate** | **Lower** | **Upper** |
| *a) Selection differentials* |  |  |  |  |  |  |  |  |  |  |  |
| Development time | 0.183 | -0.124 | 0.504 |  | **-0.230** | **-0.353** | **-0.106** |  | **-0.248** | **-0.433** | **-0.090** |
| Development time^2^ | 0.088 | -0.086 | 0.324 |  | -0.062 | -0.143 | 0.033 |  | -0.060 | -0.155 | 0.033 |
|  |  |  |  |  |  |  |  |  |  |  |  |
| *b) Selection gradients* |  |  |  |  |  |  |  |  |  |  |  |
| Development time | 0.215 | -0.061 | 0.492 |  | **-0.183** | **-0.306** | **-0.061** |  | **-0.208** | **-0.378** | **-0.044** |
| Plant size | **0.854** | **0.593** | **1.197** |  | **0.299** | **0.169** | **0.420** |  | **0.232** | **0.034** | **0.484** |
| Development time^2^ | 0.116 | -0.040 | 0.317 |  | -0.026 | -0.109 | 0.071 |  | -0.077 | -0.201 | 0.024 |

**References:** Stinchcombe, J. R., A. F. Agrawal, P. A. Hohenlohe, S. J. Arnold, and M. W. Blows. 2008. Estimating nonlinear selection gradients using quadratic regression coefficients: double or nothing? Evolution 62:2435–2440.
